# Investigating the Potential Effects of Medical AI Systems on Physician Autonomy: Pretest of a Semistructured Qualitative Interview Guide

**DOI:** 10.2196/93035

**Published:** 2026-07-16

**Authors:** John Grosser, Jule Sauerbach, Rainer Borgstedt, Sebastian Rehberg, Juliane Düvel

**Affiliations:** 1 Department of Health Economics and Healthcare Management, School of Public Health, Bielefeld University, Universitätsstraße 25, Bielefeld, North Rhine-Westphalia, 33615, Germany, 49 521-106-86319; 2 School of Public Health, Bielefeld University, Bielefeld, North Rhine-Westphalia, Germany; 3 Department of Anaesthesiology, Intensive Care, Emergency and Pain Medicine, Campus Bielefeld-Bethel, University Hospital Bielefeld, Bielefeld, North Rhine-Westphalia, Germany; 4 Centre for ePublic Health Research, School of Public Health, Bielefeld University, Bielefeld, North Rhine-Westphalia, Germany

**Keywords:** artificial intelligence, clinical decision support system, physicians, professional autonomy, pretest, qualitative research

## Abstract

**Background:**

AI is an increasingly prominent feature of contemporary health care, with medical AI systems beginning to support diagnostic and therapeutic processes in many clinical domains. Alongside the anticipated benefits of these technologies, their introduction also raises broader questions about how clinical work and professional roles may change. In particular, medical AI systems may affect physician autonomy, a key factor influencing the acceptance and long-term implementation of new medical technologies.

**Objective:**

The aim of this study was to develop and pretest a semistructured interview guide concerning the potential effects of medical AI systems on physician autonomy.

**Methods:**

The interview guide was theoretically grounded in a 7-component model of physician autonomy proposed by Schulz and Harrison. Semistructured qualitative interviews were conducted with a sample of 7 hospital physicians. Interview recordings were transcribed and analyzed using a hybrid inductive-deductive thematic approach: themes were first identified inductively from participant responses and subsequently mapped onto the 7-component model of physician autonomy proposed by Schulz and Harrison. Data were analyzed to assess both the potential effects of medical AI systems on physician autonomy and the methodological adequacy of the interview guide.

**Results:**

Most participants did not express strong concerns about losing clinical autonomy through the introduction of AI systems. However, several autonomy-related risks were identified, including potential deskilling, automation bias, limited system explainability, and increasing economic or cost-related pressures. Participants emphasized that AI should serve as a supportive tool rather than a substitute for physician judgment. All physicians agreed that AI systems should not replace clinicians as primary clinical decision-makers.

**Conclusions:**

Medical AI was largely viewed as compatible with physician autonomy; however, participants highlighted important risks that warrant attention in future research and system design. Our preliminary findings suggest that autonomy-related concerns extend beyond the direct loss of decision-making authority and include broader professional, cognitive, and organizational dimensions. However, our inductively identified themes and subthemes did not fully reflect all components of physician autonomy, indicating the need for further refinement of how to assess physician autonomy in qualitative research.

## Introduction

Medical AI systems, which range from AI-enabled decision support systems to large language models (LLMs) and agentic systems, are gaining increasing use in medical specialties ranging from oncology and pulmonology to radiology [1,2]. An important predictor of acceptance and adoption of such systems is physician autonomy [2,3], with recent reviews finding that the (perceived) risk to physician autonomy is a barrier to the acceptance of medical AI systems [4-6].

Correspondingly, recent years have seen an increase in empirical research on the effects of such systems on physician autonomy. However, this research often includes physician autonomy only implicitly or only as a secondary consideration and rarely uses a comprehensive model of physician autonomy [7]. Although there is currently no consensus model of physician autonomy, one existing approach is the 7-component model by Schulz and Harrison [8], which covers both clinical and nonclinical components of physician autonomy and has been used repeatedly in the literature [9,10]. However, this model has not yet been comprehensively applied to medical AI systems.

Our objective in this study was therefore to pretest an initial semistructured interview guide covering the effects of medical AI on all 7 components of physician autonomy. This objective gives rise to 2 research questions: a substantive (RQ1) and a methodological (RQ2).

RQ1: What are the potential effects of medical AI on physician autonomy?

RQ2: Was the interview guide able to capture all aspects of physician autonomy?

Of these, the methodological research question is the main focus of this paper, while our results concerning the substantive research question are preliminary and exploratory in nature. Based on our results, we derive a number of considerations for the further development and refinement of the initial interview guide and for future research into the effect of medical AI systems on physician autonomy.

## Methods

### Design of the Initial Interview Guide

The theoretical foundation for our initial interview guide was the 7-component model of physician autonomy proposed by Schulz and Harrison [8]. This model covers 4 clinical components and 3 nonclinical components of physician autonomy, shown in Tables1  2, which also provide examples of general limitations on physician autonomy for each component, as provided by Schulz and Harrison [8], as well as examples for potential harms and benefits identified in a recent scoping review [7].

**Table 1.** Clinical components of physician autonomy.

Component	Examples of limitations and medical AI-related benefits/harms
Acceptance of patients	Potential limitations on physician autonomy include compelling physicians to accept or reject certain patients based on geography, medical specialty, or insurance status.
Control over diagnosis and treatment	Potential limitations on physician autonomy include individual and aggregate constraints on tests or prescription costs, preset budgets, enforcement of clinical protocols, and gatekeeping. Potential benefits of medical AI for physician autonomy include enhanced decision certainty or inspiration and enhanced physician expertise. Potential harms of medical AI for physician autonomy include a loss of clinical decision-making autonomy or the loss of expertise through overreliance and automation bias.
Control over the evaluation of care	Potential limitations on physician autonomy include peer review, medical audit systems, and comparative information on care outcomes. Potential benefits of medical AI for physician autonomy include patient approval of physicians’ AI use. Potential harms of medical AI for physician autonomy include liability issues for physicians based on AI decisions or recommendations and the use of AI systems as post hoc auditing tools, as well as patient disapproval or mistrust.
Control over other professionals	Potential limitations on physician autonomy include limitations on physicians’ ability to directly manage other health professionals or include precise instructions in referrals for diagnosis or therapy. Potential benefits of medical AI for physician autonomy include the opportunity for physicians to increase their status or prestige using AI systems and the expansion of interprofessional collaboration with nonclinical professionals. Potential harms of medical AI for physician autonomy include a possible loss of status or prestige for physicians in general, a loss of physicians’ authority over other clinical professionals, or pressure by peers and superiors to adopt AI systems.

**Table 2.** Nonclinical components of physician autonomy.

Component	Examples of limitations and medical AI-related benefits/harms
Choice of specialty and practice location	Potential limitations on physician autonomy include market restrictions, bureaucratic restrictions, and educational restrictions. Potential harms of medical AI for physician autonomy include the replacement of certain specialties (or physicians, in general) by AI systems.
Control over earnings	Potential limitations on physician autonomy include workload controls, fee schedules, reimbursement rates, salaried status, and control over permitted earnings.
Control over the nature and volume of medical tasks	Potential limitations on physician autonomy include hierarchical management, contractual obligations, and the need to share scarce resources. Potential benefits of medical AI for physician autonomy include AI-based efficiency increases through the handling of administrative tasks, freeing up time for other activities. Potential harms of medical AI for physician autonomy include a loss of efficiency due to the time and effort required for data input, error correction, and training.

As is apparent from the tables, however, our previous scoping review found that existing qualitative research on the effects of medical AI systems on physician autonomy covers only a subset of the 7 components proposed by Schulz and Harrison [8]. Therefore, our initial interview guide was explicitly designed with the aim of covering all 7 components.

### Study Sample and Medical AI System Used

Our study sample consisted of 7 hospital physicians (6 men and 1 woman, including specialists, physicians with the intensive care medicine subspecialty, and senior physicians), who were recruited from 2 German hospitals that participated in the KINBIOTICS project [11,12]. Funded by the German Federal Ministry of Health, the KINBIOTICS project (“AI-based decision support for antibiotic therapy”) aimed to develop and validate AI-based algorithms that leverage a large, comprehensive dataset to support clinical decision-making and enhance the selection of suitable antibiotic therapies for patients with sepsis.

Physicians were eligible for recruitment if they had firsthand experience with the AI-based clinical decision support system (CDSS) for antibiotic prescribing evaluated in the KINBIOTICS project. This experience is a key advantage over studies that merely use vignettes to describe hypothetical AI systems to participants without any AI experience [7]. No other eligibility criteria for interview participants (eg, age, gender, and years of experience) were defined. Participants were recruited using convenience sampling as well as chain referral sampling and were approached by email. No participants withdrew from the study.

### Data Collection

We conducted semistructured qualitative interviews with these 7 physicians using the initial interview guide, which focused on (but was not limited to) participants’ experiences with the KINBIOTICS AI CDSS. As this was the first pretest of the interview guide, no prior pilot testing was performed. Interviews were conducted remotely via Zoom by one author (JS) in July and August of 2024 and were recorded using the Zoom platform’s built-in tools. The interviews were conducted in German. The duration of the interviews ranged from 14 minutes to 34 minutes, with a median duration of 21 (IQR 16-24) minutes. As the interviews were conducted online, there was no one else present besides the participants and researchers. No field notes were taken during the interviews beyond the recorded transcripts, no repeat interviews were conducted, and neither the transcripts nor the findings were returned to participants for comment.

Some members of the research team had prior relationships with the participants, in the sense that all participants also participated in the KINBIOTICS study, in which the research team was also involved. However, the researcher who conducted the interviews (JS) did not have a previous relationship with the participants. Participants were informed about the interviewer and the purposes of the research via the informed consent documents provided to them in advance.

### Data Analysis

The data were prepared and analyzed in a 5-step process based on content analysis. First, 1 author (JS) transcribed the interview recordings. Second, 2 authors (JG and JS) independently read and paraphrased the transcripts for each of the 7 interviews individually using MAXQDA (VERBI Software GmbH). This was then verified by a third author (JD). Third, 2 authors (JG and JD) jointly compared and merged these paraphrases across interviews to inductively identify subthemes emerging from the participant responses. In this step, we only considered subthemes that appeared in multiple interview transcripts. Fourth, 2 authors (JG and JD) jointly compared and merged these subthemes to form overarching themes. Within each overarching theme, we distinguished subthemes both by content (ie, different subthemes cover different aspects of the main theme) and by valence (ie, different subthemes represent different opinions or opposing views on the theme). The resulting themes and subthemes were verified by all authors. Fifth, one author (JG) attempted a mapping of the identified subthemes onto the 7 components of physician autonomy proposed by Schulz and Harrison [8], which was also verified by all authors. At each stage, disagreements were resolved by discussion and consensus among the authors. Because the coding process was conducted collaboratively, we did not analyze the intercoder reliability of our code assignments. All quotes used in this paper to illustrate the identified subthemes were translated from the original German by a native speaker of both German and English (JG). Data were collected, analyses were conducted, and the results were reported in accordance with the COREQ (Consolidated Criteria for Reporting Qualitative Research) checklist [13] (Checklist 1).

### Ethical Considerations

The overarching KINBIOTICS project received ethics approval from the Ethics Committee of the Medical Association of Westphalia-Lippe and the Westphalian Wilhelms University of Münster (2021‐699-f-S). Our qualitative interview substudy on physician autonomy received additional ethics approval from the Ethics Committee of Bielefeld University (number EUB-2024‐147). All 7 physicians provided informed consent to participate in the KINBIOTICS project and the interview substudy.

### Research Team and Reflexivity

The research team consisted of 5 authors: 2 women (JD and JS) and 3 men (JG, RB, and SR). JG has an MS in Mathematics, an MA in Bioethics and Medical Humanities, and a BA in Political Science and Sociology. He is a researcher at Bielefeld University’s Department of Health Economics. JS has an MS in Public Health and a BA in Health Data and Digitalization. At the time of the study, she was employed at BZF-Essen, a higher education institution focused on the education and professional development of adults. JD has a PhD, an MS and BS in Public Health. She is the head researcher at Bielefeld University’s Center for electronic Public Health Research. SR is a medical doctor (Dr med) and a Professor at the Medical School OWL. He is the director of University Hospital Bielefeld’s Department of Anesthesiology, Intensive Care, Emergency and Pain Medicine. RB is a medical doctor (Dr med). He works as an intensive medicine specialist at the Department of Anesthesiology, Intensive Care, Emergency and Pain Medicine.

The research team has prior research experience in digital and AI medicine [11,12,14-16], physician autonomy, digital ethics, and human resource ethics [7,17], as well as with qualitative methods, including content analysis, qualitative interviews, Delphi studies, and document analyses [18-20].

## Results

From the 7 paraphrased interview transcripts, we identified a total of 22 subthemes, which we grouped into eight overarching themes: (1) AI for triage/prioritization, (2) AI and resource utilization, (3) AI and physician skills/expertise, (4) AI as a tool—not a replacement, (5) AI in personnel management, (6) AI and career choices, (7) AI and physician income, and (8) AI and workplace efficiency. The subthemes for each theme are shown in Textbox 1.

Many of these results directly reflect potential harms and benefits of medical AI systems for physician autonomy. Potential harms include AI-induced cost pressures (Subtheme 2.2, Quote 1A), loss of confidence in physicians’ own decision-making (Subtheme 3.2, Quote 1B), as well as deskilling and automation bias (Subtheme 3.3, Quote 1C), as illustrated in Textbox 2.

Meanwhile, the potential benefits of medical AI systems for physician autonomy include increasing workplace efficiency (Subtheme 8.1, Quote 2A) and making more time available for patient care (Subtheme 8.2, Quote 2B), as illustrated in Textbox 3.

Some results also indicated an absence (or low likelihood) of potential harms. For example, as illustrated in Textbox 4, some participants would not question their competence due to peer disagreement with AI (Subtheme 3.1, Quote 3A), while most participants do not expect a loss of (clinical) autonomy due to AI (Subtheme 4.7, Quote 3B).

Textbox 1. **Themes and subthemes on AI and physician autonomy.**

**Theme 1: AI for triage/prioritization**

1.1. Some physicians think AI is capable of performing patient triage/prioritization

1.2. Some physicians think AI is incapable of performing patient triage/prioritization

**Theme 2: AI and resource utilization**

2.1. Most physicians expect AI to reduce costs (eg, by avoiding unnecessary procedures)

2.2. Some physicians expect that AI-based cost reductions could create cost pressures on physicians, harming autonomy

**Theme 3: AI and physician skills/expertise**

3.1. Some physicians would not question their competence due to peer disagreement with AI

3.2. A few physicians would question their competence due to peer disagreement with AI

3.3. Some physicians expect AI to lead to deskilling and/or automation bias

**Theme 4: AI as a tool—not a replacement**

4.1. Most physicians think AI is a useful decision aid

4.2. A few physicians argue that AI is only as useful as the data provided to it

4.3. A few physicians consider it important that physicians understand how AI reaches its conclusions

4.4. Some physicians think AI cannot replace physicians’ clinical experience

4.5. All physicians think AI should not replace physicians as clinical decision-makers

4.6. Some physicians argue that they are legally accountable and should therefore remain responsible

4.7. Most physicians do not expect a loss of (clinical) autonomy due to AI

**Theme 5: AI in personnel management**

5.1. Some physicians think AI can be a useful tool in personnel management (eg, assigning work schedules)

5.2. Most physicians think AI should not replace (senior) physicians in personnel management

**Theme 6: AI and career choices**

6.1. Some physicians would consider AI a positive factor in future career choices

6.2. Some physicians would consider AI a neutral factor in future career choices

**Theme 7: AI and physician income**

7.1. Most physicians expect AI to have no meaningful impact on their income

7.2. A few physicians expect AI to have a meaningful impact on their income

**Theme 8: AI and workplace efficiency**

8.1. All physicians expect AI to save time and increase workplace efficiency

8.2. Some physicians expect AI-based efficiency gains to make more time available for patient care

Textbox 2. **Subthemes on potential AI harms for physician autonomy.**

**Quote 1A (Subtheme 2.2):**

“Hospital administrators always try to work economically and avoid [economic] losses. I would imagine health insurance providers would likely advocate for listening to AI. One needs to be sure that this will not go the wrong way.”

**Quote 1B (Subtheme 3.2):**

“If I were to say ‘the patient has this [a certain diagnosis] and we should implement that [a certain treatment]’ and the AI says something different, that would definitely influence me […] because it would make me feel uncertain.”

**Quote 1C (Subtheme 3.3):**

“I could imagine […] that people might stop reasoning for themselves, questioning things and thinking about […] the right therapy or diagnosis […] that one relies too much [on the AI].”

Textbox 3. **Subthemes on potential AI benefits for physician autonomy.**

**Quote 2A (Subtheme 8.1):**

“I spend […] a lot of time looking at, summarizing and interpreting data. If there were an AI that looks at data independently and makes me aware of problems without me having to look for the problems myself, that would definitely be a significant time saver.”

**Quote 2B (Subtheme 8.2):**

“Every doctor spends a large amount of their time with administrative tasks. An AI system can surely reduce doctors’ workloads, so that they can concentrate more on the patients.”

Textbox 4. **Subthemes on absent or unlikely AI harms for physician autonomy.**

**Quote 3A (Subtheme 3.1):**

“The experiences and values I have, no AI system can reach. Because of that, it [peer disagreement with AI] would not make me question myself.”

**Quote 3B (Subtheme 4.7):**

“I’m not worried about a loss of autonomy. I can say that very clearly.”

Still other results referred to participants’ views on AI and physician autonomy without enumerating specific potential harms and benefits, instead offering recommendations related to the use of medical AI systems. For example, all participants agreed that AI should not replace physicians as clinical decision-makers (Subtheme 4.5, Quote 4A), with some arguing that physicians are legally accountable and should therefore remain responsible (Subtheme 4.6, Quote 4B). Most participants also agreed that AI should not replace (senior) physicians in their personnel management functions (Subtheme 5.2, Quote 4C), while a few highlighted the importance of physicians understanding how medical AI systems reach their conclusions (Subtheme 4.3, Quote 4D). These subthemes are illustrated by the quotes in Textbox 5.

In contrast, however, half of the identified subthemes did not relate to physician autonomy at all or did so only indirectly. Of these, 7 subthemes concern the capabilities of medical AI systems. In particular, participants were split as to whether AI is capable of performing triage/prioritization (Subthemes 1.1 and 1.2, Quotes 5A and 5B) and mostly saw AI as capable of reducing health care costs (Subtheme 2.1, Quote 5C). Some considered AI to be a useful tool for personnel management (Subtheme 5.1, Quote 5D). For clinical decision-making, they considered AI a useful decision aid (Subtheme 4.1, Quote 5E) that is reliant on appropriate, high-quality data (Subtheme 4.2, Quote 5F) and cannot fully replace the clinical experience of a real physician (Subtheme 4.4, Quote 5G). These subthemes are illustrated by the quotes in Textbox 6.

The remaining 4 subthemes are also not directly related to physician autonomy, as illustrated in Textbox 7. In particular, Subthemes 6.1 (Quote 6A) and 6.2 (Quote 6B) concern whether the presence of medical AI systems would influence participants’ future career choices, in the sense that participants may be more or less likely to want to work in an environment in which such systems are used. This is, however, distinct from physicians’ control over their choice of specialty and practice location (as a component of physician autonomy). Meanwhile, Subthemes 7.1 (Quote 6C) and 7.2 (Quote 6D) concern the potential effect of AI on physicians’ income. This is, again, distinct from physicians’ control over their own earnings (as a component of physician autonomy).

Textbox 5. **Subthemes on recommendations for AI use.**

**Quote 4A (Subtheme 4.5):**

“In the end, what’s important is that the final decision stays with the doctor and is not replaced by the AI.”

**Quote 4B (Subtheme 4.6):**

“I think that [the fact that doctors will remain responsible for clinical decisions] is related to liability law. In the end, the doctor must be liable for their decisions.”

**Quote 4C (Subtheme 5.2):**

“The important thing is that one has the final word [concerning the duty schedule]. So, despite the AI, in the end it’s the doctor or the [person responsible for the duty schedule] who makes the decision.”

**Quote 4D (Subtheme 4.3):**

“The key point is that you can understand the foundation for the AI’s decisions.”

Textbox 6. **Subthemes on AI capabilities.**

**Quote 5A (Subtheme 1.1):**

“Prioritization is all about hard facts that can be easily quantified […] In that sense, I think handling such prioritization would be a pretty good task for an AI. I would definitely trust an AI to be able to do that.”

**Quote 5B (Subtheme 1.2):**

“When it comes to triage […] to determining who gets treated first, that’s where it gets difficult, because you also have to clinically assess the patient, and an AI can’t do that; it doesn’t see the patient, it just looks at the numbers.”

**Quote 5C (Subtheme 2.1):**

“I believe AI can be used to make the diagnostic process more [economically] rational.”

**Quote 5D (Subtheme 5.1):**

“I think there’s a lot that can be done [using AI] when it comes to [duty] scheduling […] I believe it can really ease the burden of daily operational planning [of personnel].”

**Quote 5E (Subtheme 4.1):**

“I think that when used correctly, it [AI] can be very helpful, and I think that many decisions that need to be made can be made better and faster thanks to large amounts of [pre-analyzed] data.”

**Quote 5F (Subtheme 4.2):**

“It’s about whether you even have the ability to analyze the available data. That is something we lack in Germany. For example, when a patient visits a general practitioner, that patient may have already seen five other doctors. The general practitioner isn’t informed of this, nor of the findings from those previous visits. If the general practitioner had access to all this information, they might be able to use an AI system to make a better diagnosis […] AI systems thrive on data. They simply need data. And if that data isn’t available […] then things become very difficult.”

**Quote 5G (Subtheme 4.4):**

“Particularly when it comes to decisions about the end of life, I don’t think AI can make them in the same way a human can, because a human can [...] see [...] what patients and their families need in that very moment. And I think that’s simply the kind of data AI can’t capture very well.”

Textbox 7. **Subthemes on other effects of AI**

**Quote 6A (Subtheme 6.1):**

“So if there are two similar [employer] locations that I’d both be interested in, and one of them uses AI, then I’d say, ‘OK, I’ll go there.’”

**Quote 6B (Subtheme 6.2):**

“I don’t think I would switch clinics just because I know they do it [use AI] at another clinic but not at ours.”

**Quote 6C (Subtheme 7.1):**

“What doctors’ income looks like depends less on AI systems and more on whether there is a surplus or a shortage of doctors […]. I don’t believe that AI systems will fundamentally change the practice of medicine to the extent that we’d say ‘The doctor is now just a part-time doctor and the rest is done by AI and that’s why we’d pay them less.’”

**Quote 6D (Subtheme 7.2):**

“If we fully implement AI-powered, guideline-based treatment algorithms, much of what we do will no longer be necessary. I’m convinced of that, and ultimately, there’s money at stake there.”

Overall, the inductively identified subthemes directly related to physician autonomy did not cover all 7 components proposed by Schulz and Harrison [8], as demonstrated by the mapping of subthemes to components in Tables3  4. In particular, we identified no subthemes explicitly pertaining to the components “acceptance of patients,” “choice of specialty and practice location,” and “control over earnings.”

Notably, all of the potential benefits of AI on physician autonomy identified by participants were mapped to nonclinical components (in particular, control over the nature and volume of medical tasks), while all of the potential harms identified by participants were mapped to clinical components (in particular, control over diagnosis and treatment), as were responses concerning the absence of potential harms.

**Table 3.** AI and clinical components of physician autonomy.

Component	Mapped subthemes
Acceptance of patients	No subthemes mapped
Control over diagnosis and treatment	AI-induced cost pressures^a^ Loss of confidence by physicians due to peer disagreements with AI^a^ No loss of confidence by physicians due to peer disagreement with AI^b^ AI-induced deskilling and automation bias^a^ No loss of (clinical) autonomy due to AI^b^ Physicians should understand how AI reaches its conclusions^c^ AI should not replace physicians as clinical decision-makers^c^
Control over the evaluation of care	Physicians are legally accountable and should thus remain responsible^c^
Control over other professionals	AI should not replace (senior) physicians in their personnel management functions^c^

a A potential harm.

b The absence of a potential harm.

c Recommendations.

**Table 4.** AI and nonclinical components of physician autonomy.

Component	Mapped subthemes
Choice of specialty and practice location	No subthemes mapped
Control over earnings	No subthemes mapped
Control over the nature and volume of medical tasks	Increased workplace efficiency^a^ More time for patient care^a^

a Potential benefits.

## Discussion

In this paper, we report the results of a pretest of an initial semistructured interview guide for investigating the effects of medical AI systems on physician autonomy. Concerning our substantive research question, our initial results indicate that most participants did not fear a loss of clinical autonomy. Nevertheless, deskilling, automation bias, lack of explainability, and cost pressures emerged as potential AI-based risks for physician autonomy. All participants agreed that AI systems should not replace physicians as clinical decision-makers.

Our findings, while preliminary in nature, are in line with existing research on AI-CDSS and (perceived) physician autonomy. Consistent with a recent systematic review of trust and perceptions in AI-CDSS [21], physicians generally do not report an immediate fear of losing clinical autonomy, even when interacting with complex AI-driven systems. Instead, physicians tend to view AI as an assistive tool whose primary value lies in supporting and augmenting, rather than replacing, clinical judgment.

Furthermore, a recent mixed methods review [22] suggests that an increasing reliance on AI-based decision support systems could gradually reduce opportunities to maintain and exercise clinical skills, particularly in diagnostic reasoning. This frames deskilling as a long-term risk rather than a direct consequence of system use.

Similar concerns are raised in narrative and perspective-based publications, which caution that overreliance on AI recommendations may disproportionately affect less experienced clinicians and subtly reshape professional expertise over time [23]. Consistent with our preliminary findings, existing research thus conceptualizes deskilling as a conditional and future-oriented risk rather than an already realized loss of autonomy.

Our participants identified the risk of automation bias, which is also supported by existing research. Studies demonstrate that clinicians can exhibit automation bias when they are inclined to follow erroneous AI recommendations and that task context, time pressure, and confidence in the system can influence this effect [24,25].

Studies on explainability in AI-CDSS highlight that opaque “black-box” models can compromise clinicians’ ability to understand and justify recommendations, potentially undermining their sense of epistemic control, a core component of perceived autonomy in clinical reasoning [26,27]. Some studies go further, noting that explainability can paradoxically both enhance trust and heighten perceptions of professional identity threat, depending on how system design features influence the clinician’s role in decision workflows [28].

Our participants also foregrounded organizational and economic pressures as contextual moderators of autonomy perceptions. This is corroborated by broader research showing that physicians’ experiences of AI in practice are not solely shaped by technology per se but by institutional demands, workflow integration, and resource constraints highlighted in implementation literature and systematic reviews of AI in clinical contexts. Finally, the unanimous agreement among our participants that AI should not replace physicians as clinical decision-makers aligns with prior empirical and normative research. Clinicians consistently emphasize the preservation of human responsibility, accountability, and the clinician-patient relationship, while acknowledging the supportive role AI can play in performing clinical tasks [29,30].

These findings concerning our substantive research question are, however, preliminary and exploratory in nature. In particular, our substantive findings are derived from a small sample of physicians and based purely on qualitative, not quantitative, analysis. Additionally, our sample included mostly men (only one woman), which further undermines the generalizability of our substantive results. Furthermore, our research was conducted in Germany, and our preliminary findings reflect the nature of the German health system (eg, Quote 6C). The generalizability of these findings is likely further limited by the fact that we recruited participants from only 2 hospitals, which creates the possibility that our results reflect contextual factors specific to those hospitals. For example, different participants being embedded in similar peer networks and administrative structures could have shaped their perceptions of both their own autonomy as well as the role and capabilities of medical AI systems.

Finally, our substantive findings are based on participants’ experiences with a single AI system (KINBIOTICS), an AI CDSS for antibiotic prescribing. Although this experience is an advantage over studies based purely on vignettes or hypotheticals, our results may not generalize to other forms of medical AI systems, including LLMs [31] and AI agents [32].

Use cases for medical LLMs have some overlap with use cases for AI CDSS. In particular, LLMs for clinical workflow support are designed to reduce administrative burdens and can propose plausible diagnostic options, serving as secondary references or clinical reasoning aides [31], a role similar to many AI CDSS. However, LLMs present novel risks to physician autonomy. For example, LLMs may appear to be more explainable (ie, less opaque) than AI CDSS as they are able to generate plausible-sounding explanations and justifications of their decisions and recommendations. These explanations, however, may be hallucinated (ie, fluent linguistically but incorrect, ungrounded, or misleading factually) [31,33] and generally do not reflect a “true” underlying reasoning process. Furthermore, LLMs generating persuasive responses and explanations using natural language may increase the risk of physician overreliance on AI systems.

Agentic AI systems also present novel risks to physician autonomy beyond those posed by AI CDSS and LLMs. Unlike LLMs, AI agents exhibit goal-directed behavior, persistent longitudinal memory, and the ability to take independent action, rather than merely offering text-based recommendations [32]. These capabilities mark key changes in the physician-AI relationship concerning oversight and delegation of clinical tasks to AI agents, raising important concerns for physician autonomy, including unclear accountability between physicians and AI, as well as reduced physician control over clinical workflows.

To address these concerns, future research should (1) recruit larger, more diverse, and generalizable samples, (2) use quantitative methods in addition to qualitative methods, and (3) explicitly investigate the similarities and differences in the effects of different AI systems (including CDSS, LLMs, and agentic systems) on physician autonomy. In particular, given the novel status of many currently available medical AI systems, future research should focus on the long-term effects of these different types of AI on physician autonomy.

Concerning our methodological research question, our inductively identified themes and subthemes did not fully reflect all 7 components of physician autonomy. In particular, our results did not cover the effect of medical AI systems on physicians’ control over their earnings, their acceptance of patients, or their choice of specialty and practice location. This result is surprising, since our study was conceived with the goal of covering all 7 components of physician autonomy. Its failure to do so is likely due to a combination of several problems with the initial interview guide.

First, for 2 of the missing components (choice of specialty and practice location and control over earnings), the prompts in the initial interview guide generated responses that were not directly related to physician autonomy. In particular, participant responses to these prompts concerned whether they would consider the presence of AI as a positive factor in their future career decisions and whether they expect AI to have a meaningful effect on their future income. This diverges from what we were actually interested in concerning these components: the ways in which AI could potentially constrain or enable physicians’ *ability to choose their specialty/practice location* and their *ability to control their own earnings*. This problem may indicate that we did not sufficiently explain these components of physician autonomy to participants during the interviews.

Relatedly, many subthemes emerging from participant responses referred to the overall promise of medical AI systems (ie, the ability or inability of AI to perform various tasks), rather than the effect of these systems on physician autonomy. This indicates that the interview guide did not make it sufficiently clear that we were interested specifically in the effects of medical AI on physician autonomy, not on clinical practice in general.

In their responses, participants also switched back and forth between discussing the effects of the concrete medical AI system introduced in the KINBIOTICS project, on the one hand, and discussing other hypothetical AI systems and their effects, on the other hand. This problem is compounded by the fact that our participants had limited experience with medical AI systems outside the KINBIOTICS project.

These concerns give rise to a number of considerations that should be addressed in physician autonomy research. In particular, in addition to explicitly addressing all components of physician autonomy, researchers should (1) make it clear to the participants that the outcome of interest is the effect of medical AI on physician autonomy (rather than its potential to generate other clinical benefits), (2) more clearly explain each of the components of physician autonomy to the participants, and (3) make it explicit whether responses should refer to a specific medical AI system with which participants have experience, to a specific hypothetical medical AI system, or to medical AI systems in general. When investigating medical AI systems in general (rather than evaluating a specific real-world system), researchers should take care to distinguish the effects of different kinds of medical AI systems (eg, CDSS, LLMs, and agentic systems) on physician autonomy by including questions tailored to each system type. For example, research on LLMs should include questions that address the natural language and conversational capabilities of such models, while research on agentic systems should reflect their capability to take independent action.

Our results may also point toward a deeper issue with our approach: does the 7-component model of physician autonomy as proposed by Schulz and Harrison [8] actually correspond to physicians’ own conception of what physician autonomy means to them? In other words, do physicians actually see control over earnings or the choice of specialty and practice location as part of their autonomy as physicians? Thus, more basic research into the nature and significance of physician autonomy to physicians may be needed before the effect of medical AI systems on physician autonomy can be properly investigated and understood. In particular, Delphi methods, which have been used to generate conceptual models on, for example, task shifting [34], technology adoption [35], and physician empathy [36], could be used to develop a conceptual framework of physician autonomy that reflects the perspectives and experiences of physicians. Furthermore, the extent to which different groups of physicians in different settings and contexts consider different components of such a framework to be part of their physician autonomy, as such, could be investigated using comparative quantitative surveys.

Finally, beyond individual perceptions of autonomy, ethical analyses emphasize that physician autonomy in clinical practice is inherently relational, grounded in the clinician’s ongoing interaction with patients and in shared decision-making processes rather than in isolated decision authority [37]. AI-based decision support systems, by introducing a third actor into the traditional physician-patient dyad, may reconfigure this relational autonomy, not by overtly displacing physicians’ authority but by altering communicative and interpretive dynamics in clinical encounters. This perspective suggests that autonomy concerns cannot be fully understood without considering how AI shapes the physician-patient relationship and responsibilities in health care.

## Supplementary material

10.2196/93035 Checklist 1 COREQ checklist.

## Abbreviations

CDSS: clinical decision support system
COREQ: Consolidated Criteria for Reporting Qualitative Research
LLM: large language model
